# Genome-Wide Analysis of the Phospholipase D Family in Five Cotton Species, and Potential Role of *GhPLD2* in Fiber Development and Anther Dehiscence

**DOI:** 10.3389/fpls.2021.728025

**Published:** 2021-10-01

**Authors:** Changkai Ma, Qian Zhang, Jiaoyan Lv, Kaikai Qiao, Shuli Fan, Qifeng Ma, Chaojun Zhang

**Affiliations:** ^1^ State Key Laboratory of Cotton Biology, Institute of Cotton Research of CAAS, Key Laboratory of Cotton Genetic Improvement, Ministry of Agriculture, Anyang, China; ^2^ Zhengzhou Research Base, State Key Laboratory of Cotton Biology, Zhengzhou University, Zhengzhou, China

**Keywords:** phospholipase D, cotton fiber, anther dehiscence, male sterility, *GhPLD2*

## Abstract

Phospholipase D (PLD) and its hydrolysis product phosphatidic acid play an important role in the regulation of several cellular processes, including root growth, pollen tube elongation, and microtubule reorganization. Here, we systematically identified and analyzed the membership, characterization, and evolutionary relationship of PLDs in five species of cotton. The results of the transcriptomic analysis suggested that the evaluated *PLD* genes showed high expression levels in anther tissue and during the fiber initiation and elongation periods. Quantitative real-time polymerase chain reaction showed differential expression of *GhPLD* genes in the anthers of *photoperiod sensitive male sterility mutant 5* (*psm5*). Previous research on multiple stable quantitative trait loci also suggests the role of *PLD* genes in the fiber development. Further analyses showed that GhPLD2 protein is localized to the plasma membrane. The virus-induced gene silencing of *GhPLD2* in cotton seedlings repressed its expression by 40–70%, which led to a reduction in reactive oxygen species (ROS) levels, 22% anther indehiscence, and disrupted fiber initiation and elongation. Thus, we inferred that GhPLD2 may promote ROS production, which, in turn, may regulate anther dehiscence and fiber development.

## Introduction

Globally, cotton (*Gossypium* spp.) is among the most important cash crops for textiles, fine chemical raw materials, and strategically important supplies. Among the 50 cotton species, four are cultivated species, namely *Gossypium hirsutum* L. [(AD)_1_, 2*n*=4*x*=52], *G. arboreum* L. [(AD)_2_, 2*n*=4*x*=52], *G. barbadense* L. (A_1_, 2*n*=4*x*=26), and *G. herbaceum* L. (A_1_, 2*n*=4*x*=26; Wendel and Grover, 2015). Among them, upland cotton (*G. hirsutum* L.) accounts for more than 95% of cotton production owing to its excellent yield potential and adaptability. Examining the male sterile lines provides an important theoretical basis for understanding the breeding of hybrid cotton, the yield of which is about 15% higher than that of conventional cotton, and has significantly improved fiber quality.

Successful anther dehiscence, or maturation of stamens, depends on several processes, including dilation of the endothecium layer, deposition of fibrous bands in endothecial cells, degradation of septal cells, and opening of the stomium (Keijzer, 1987). The process of cotton fiber initiation involves cell wall loosening due to increased turgor pressure in selected epidermal cells. Subsequently, the cotton fiber undergoes: (a) polarity elongation; (b) transitional wall thickening and primary wall remodeling; and (c) secondary wall thickening (Haigler et al., 2012). Cell division is a vital process for anther dehiscence and fiber development, and the previous evidence suggests that reactive oxygen species (ROS) homeostasis is critical for the maintenance of normal cell division in the root-tip cells of *Triticum turgidum* and *Arabidopsis thaliana* (Livanos et al., 2012b). *ANTHER DEHISCENCE REPRESSOR* (*ADR*) suppresses ROS accumulation and endothecium thickening negatively regulates anther dehiscence by suppressing ROS accumulation and endothecium thickening in *A. thaliana* (Dai et al., 2019). Thus, phospholipase D (PLD), as a an activator of ROS production, may play an important role in this process (Livanos et al., 2012a).

Phospholipase D is a lipid hydrolase of the phospholipase superfamily and is widely found in prokaryotes and eukaryotes. Based on the distinct protein domain identities found in the N-terminal with specific catalytic properties, the PLD family can be divided into the C2-PLD, PX/PH-PLD, and SP-PLD subfamilies. The C2 domain is a Ca^2+^- and phospholipid-binding domain; the PX and PH domains consist of the phox homologue and the pleckstrin homologue, respectively, which are two distinct phosphoinositide-interacting structural folds; SP is a signal peptide (Wang, 2005; Li et al., 2007). Depending on gene structures, sequence similarities, conservative domains, and biochemical properties, PLDs in plants can be further classified into six classes in plants: α, β/γ, δ, ε, ζ, and φ. Among these, five classes (α, β/γ, δ, ε, ζ) have been identified in *Arabidopsis*, and PLDφ is found in rice (Qin and Wang, 2002; Li et al., 2007). The phospholipid-hydrolyzing activities of PLDα, PLDβ, PLDγ, PLDδ, and PLDε are calcium-dependent (C2-PLD), while that of PLDζ is calcium-independent (PX/PH-PLD). Unlike the C2 domain or PX and PH domains at the N-terminus, the PLDφ harbors a signal peptide and is therefore designated as SP-PLD.

Currently, *PLD* genes have been identified in various plant species, such as *A. thaliana* (Eliáš et al., 2002; Qin and Wang, 2002), *Oryza sativa* L. (Li et al., 2007), *Populus alba* L., *Vitis vinifera* L. (Liu et al., 2010), *Glycine max* L. (Zhao et al., 2012), *Malus domestica* B., *Fragaia ananassa Duchesne*, *Prunus salicina* L. (Du et al., 2013), *Prunus persica* L. (Wan et al., 2019), *Zea mays* L. (Chen et al., 2017), *Brassica napus* L. (Lu et al., 2019), *G. arboretum* (Tang et al., 2016b), *G. hirsutum*, and *G. raimondii* (Tang et al., 2016a). In this study, we analyzed the PLD gene family in *G. barbadense* and *G. herbaceum* for the first time and re-analyzed them with new genomic data of *G. hirsutum*, *G. arboreum*, and *G. raimondii*. We also examined a newer male-sterile cotton mutant, *photoperiod sensitive male sterility mutant 5* (*psm5*) to investigate the potential function of the *PLD* gene in anther development. *psm5* is a filial generation of *psm4* and W10 having defective anther dehiscence, to discover the potential function of the *PLD* gene in anther development (Zhang, 2018). The functions of the candidate gene *GhPLD2* in fiber development and anther dehiscence were also investigated.

## Materials and Methods

### Plant Materials

Upland cotton (*G. hirsutum* cv. W10) and *psm5*, generated by self-pollination of hybrid F_1_ of *psm4* was crossed with W10 (Zhang, 2018), were cultivated under 16h light/8h darkness in a culture room at approximately 26–28°C. Samples of W10 and *psm5* anther were collected during two sampling periods: the binuclear period (flower bud length: 10mm) and a day before the flowering (flower bud length: 30mm). All of these samples were immediately frozen in liquid nitrogen and thereafter stored at −80°C until RNA extraction. Each sample comprised pooled material from 20 cotton plants. Three independent biological experiments were performed for each sample.

### Identification of PLD Gene Family in Cotton

The PLD sequences of five *Gossypium* species were obtained from CottonFGD (Zhu et al., 2017)^1^ and COTTONGEN (Yu et al., 2014).^2^ A hidden Markov model atlas of PLDc (PF00614) and PLDc_2 (PF13091) was used to find the PLD proteins in the *Gossypium* spp. database. Twelve AtPLD protein sequences, identified from TAIR,^3^ were used as queries in the cotton genome database with the Basic Local Alignment Search Tool. In addition, the number of members of PLD gene family in five cotton species was checked by BLAST analysis of the reference genome sequence itself. Pfam,^4^ and the simple modular architecture research tool,^5^ and the National Center for Biotechnology Information conservative domain database^6^ were used to verify the presence of two complete HKD domains in the putative PLD candidates. Proteins with incomplete domains were then manually removed. The theoretical molecular weight (Mw) and isoelectric point (pI) were computed by ExPASy.^7^ The presence of a signal peptide was predicted using the CPHmodels 3.2 Server.^8^

### Bioinformatics Analyses

Multiple sequence alignment of the PLD protein sequences in *Gossypium* spp. and *Arabidopsis* genomes was performed with the E-INS-I algorithm of MAFFT v. 7.205 (Katoh and Standley, 2013). The best-fitting substitution model was estimated using AIC in jModelTest v. 2.1.2 (Darriba et al., 2012). Maximum likelihood (ML) and Bayesian inference (BI) were performed at the CIPRES Science Gateway (Miller et al., 2011) using RAxML 8.1.12 (Stamatakis et al., 2008) and MrBayes 3.2.6 (Ronquist et al., 2012), respectively. ML analysis included 1,000 bootstrap replicates under the LG+I+G model. BI analysis was made using the GTR+I+G model, analyses were run for 5×10^6^ generations and Markov chains were sampled every 100 generations and 25% of the converged runs were regarded as burn-in.

The Multiple Collinearity Scan toolkit was used to ascertain the collinearity relation between the PLD members. The program KaKs_Calculator was used to calculate the nonsynonymous substitution rate (Ka) and synonymous substitution rate (Ks) of paralogous *PLD* genes. The model averaging method was used to calculate the values of Ka and Ks.

### Co-localization of PLDs With Stable QTLs for Fiber Development

Quantitative trait loci (QTLs) and their corresponding molecular markers were investigated using COTTONGEN (see Footnote 2). The QTLs for fiber development-related traits were identified (Yu et al., 2014). To acquire the information on the physical position, first, the sequence of each marker was first retrieved from COTTONGEN. Second, the molecular marker sequences were aligned with the HAU genome of upland cotton using BLAST in the CottonFGD website (Zhu et al., 2017).^9^ Using Mapchart software, we constructed a diagram to display the location of *PLD* genes on chromosomes along with their adjacent loci and QTLs (Voorrips, 2002). As the cotton LD decay was approximately 0.80Mb, gene locations pinpointing to the QTL or within 500kb of a QTL were considered as anchored genes in QTL analysis.

### Expression of *GhPLD* Genes

To investigate the potential biological functions of PLDs, the expression patterns of diverse *PLD* genes in upland cotton were examined based on the RNA-seq data from the NCBI Sequence Read Archive.^10^ The heat map of tissue-specific expression was plotted using the TBtools software (Chen et al., 2020a). Total RNA for quantitative real-time polymerase chain reaction (qPCR) analysis was extracted from cotton anthers using the RNAprep pure plant kit according to the manufacturer’s instructions (TIANGEN, Beijing, China). The PrimeScript®RT reagent kit (TaKaRa, Dalian, China) was for the synthesis of cDNAs by reverse transcription to synthesize the cDNAs. The primer sequences for qPCR are listed in Supplementary Table S3. The qPCR data were processed with the 2^−ΔΔ*C*T^ method (Livak and Schmittgen, 2001). Three biological replicates were performed, and mean±SD values were calculated accordingly.

### Subcellular Localization of GhPLD2 Protein

The coding sequence of *GhPLD2* was cloned and ligated into the pCAMBIA-2300-35S-eGFP vector. Then, the fusion vector was transformed into *Agrobacterium tumefaciens* strain GV3101 and transiently expressed in tobacco (*Nicotiana benthamiana*) leaves. The injected tobacco leaves were cultured for 36–48h. The eGFP fluorescence in the leaves was observed using a Dmi8 inverted microscope (Leica, Wetzlar, Germany), upon labeling the cell membranes using the membrane dye FM4-64 (4mM; AAT Bioquest).

### Virus-Induced *GhPLD2* Gene Silencing in Cotton

The recombinant vector pCLCrV::GhPLD2 was constructed with the pCLCrV vector and a 200bp fragment of GhPLD2. Then, the vectors containing the recombinant vector (pCLCrV::GhPLD), the positive control vector (pCLCrV::PDS), and empty vector (pCLCrVA) were separately transformed into *A. tumefaciens* strain GV3101, respectively. The transformed bacteria were collected by centrifugation and resuspended in osmotic buffer (10mM MES, 10mM MgCl_2_, and 0.2mM acetosyringone) with a final concentration of OD_600_=1.5. Then, *A. tumefaciens* with pCLCrVB and *A. tumefaciens* with the pCLCrV::GhPLD2, pCLCrVA, and pCLCrV::PDS, plasmids, respectively, were mixed in equal amounts and incubated at 28°C for 3h; they were injected into cotyledons of W10 seedings. Subsequently, all seedlings were transferred to a greenhouse under conditions of 16h/8h (light/dark) cycle at 25°C, for culture. Total RNA from the anthers of the gene-silenced cotton plants was extracted and processed for differences in gene expression analysis.

### Determination of the Phospholipase D Activity and ROS Levels

The anthers of cotton were harvested and weighed. A specified amount of phosphate-buffered saline (pH=7.4) was added to the samples and homogenized by grinders. The samples were centrifugated for 20min at 14,000rpm; solid material was removed, and the supernatant was carefully collected. PLD activity was determined using the Plant Phospholipase D ELISA Kit (MEIMIAN) according to the manufacturer’s instructions. ROS production rate was measured by Mitochondrial ROS production rate detection Kit (Comin, Suzhou, China), and ROS levels were assayed using the Plant ROS ELISA Kit (NanJing JianCheng Bioengineering Institute, Nanjing, China) according to the manufacturer’s instructions. Three biological replicates were performed; the data for the corresponding anthers from at least two flowers were recorded from each identically treated plant.

### *In vitro* Culture of Cotton Ovules Treated With *n*-Butanol and Tert-Butanol and Quantitation of Cotton Fiber

The ovaries from flowers of W10 plants, 1day post-anthesis (DPA), were soaked in 75% ethanol for 2min, and washed with 100% ethanol for 2min. They were then dried in a sterile petri dish. Subsequently, the ovules were collected from the ovaries and were placed in a liquid BT medium with 5μM indole-3-acetic acid and 0.5μM gibberellic acid. Next, 0.2% *n*-butanol (1-But), tert-butanol (3-But), and sterile water (control) were added, respectively, for each treatment; they were cultivated in the dark at 30°C for 20days. Fiber length and total fiber units (TFUs) were measured according to the methods described in previous studies (Beasley and Ting, 1974; Gong et al., 2014).

## Results

### Molecular Structure Analysis of *PLD* Genes in *Gossypium*

**Figure 1:**
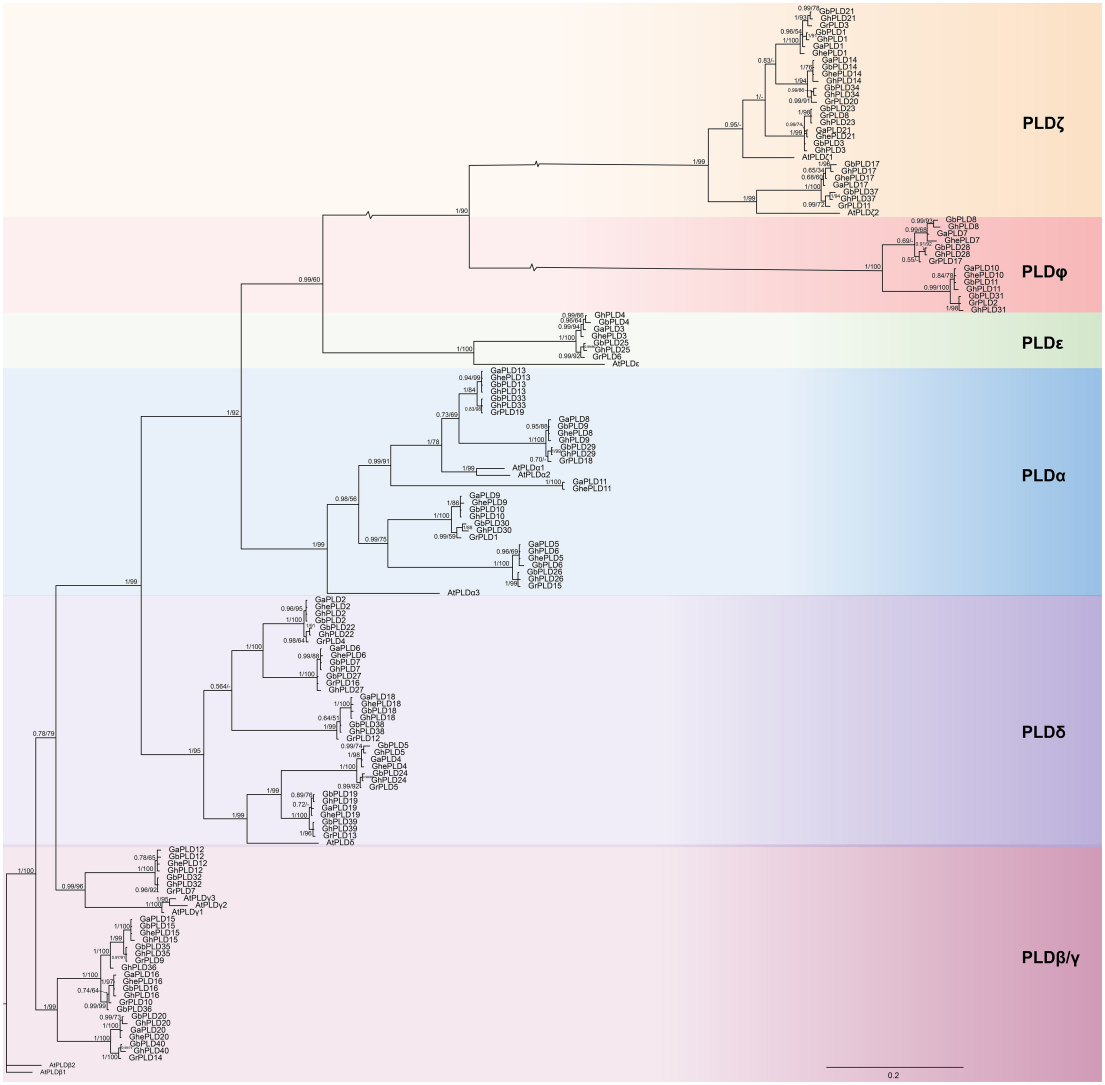
Phylogenetic tree of the phospholipase D (PLD) gene family. Molecular phylogenetic analysis of 154 PLD proteins from *Gossypium hirsutum*, *G. barbadense*, *G. herbaceum*, *G. arboreum*, *G. raimondii*, and *Arabidopsis thaliana*. Groups α, β/γ, δ, ζ, ε, and φ show the phylogenetic clusters of genes. Different groups are represented using different background colors. Numbers at nodes correspond to posterior probabilities from Bayesian analysis (left) and the bootstrap value from ML analysis (right). Scale bar indicates the rates of substitutions/site.

In this study, 40, 40, 21, 21, and 20 *PLD* genes were identified in *G. hirsutum* [(AD)_1_, 2*n*=4*x*=52], *G. barbadense* [(AD)_2_, 2*n*=4*x*=52], *G. herbaceum* (A_1_, 2*n*=4*x*=26), *G. arboreum* (A_2_, 2*n*=4*x*=26), and *G. raimondii* (D_5_, 2*n*=2*x*=26), respectively. A total of 142 PLD proteins were noted according to their chromosomal positions. Gene names, gene IDs, chromosomal locations, amino acid amounts, molecular weights, pIs, subcellular localization, and signal peptide predictions are summarized in Supplementary Table S1. To evaluate the evolutionary relationships between the *PLD* genes in *Gossypium*, a phylogenetic tree based on maximum likelihood was constructed with PLD proteins from *A. thaliana* and the five cotton species. The phylogenetic analysis showed that 154 PLDs were divided into six monophyletic clades: α, β/γ, δ, ε, ζ, and φ (Figure 1). Among the six clades, the largest group was δ comprising 36 members including 10 GhPLDs, 10 GbPLDs, five GhePLDs, five GaPLDs, five GrPLDs, and one AtPLDδ; group ε was the smallest comprising eight members, including two GhPLDs, two GbPLDs, one GhePLDs, one GaPLDs, one GrPLDs, and one AtPLDε. The details of the group compositions are listed in Supplementary Table S1. The predicted pI values of the PLDs ranged from 5.32 to 9.38. All of the PLDs in the φ clade had the signal peptide.

Patterns of intron-position correspondence between widely diverged eukaryotic species have provided valuable information into the evolutionary relationships. We analyzed the exon-intron structures of different PLDs in *A. thaliana* and five cotton species (Supplementary Figure S1). The intron numbers and lengths highly varied among PLD members. But most PLD members within the same subgroups shared similar intron/exon numbers. The result suggested PLD member are conserved in different species during evolution.

### Evolution of *PLD* Genes in *Gossypium*

To decipher the evolutionary relationships of *PLD* genes, interspecific synteny was analyzed for the five cotton species (Supplementary Figure S2). Eighteen collinear gene pairs were obtained for the D-subgenomes of two allotetraploid cotton species and *G. raimondii*. The collinearity between the A-subgenomes of the two allotetraploid cotton species and *G. herbaceum* and *G. arboreum* were also analyzed. We found that there were 16 collinear gene pairs between two allotetraploid cotton species and *G. herbaceum*; there were 17 collinear gene pairs between the two allotetraploid cotton species and *G. arboreum*. Furthermore, there were 18 collinear gene pairs between *G. hirsutum* and *G. barbadense* for both the A-subgenomes and D-subgenomes (Supplementary Figure S2). These results suggested high collinearity in allotetraploid cotton species and their corresponding A- and D-subgenomes in diploid cotton; many *PLD* genes in *G. raimondii* and the D-subgenomes of the two allotetraploid cotton species were evolutionarily closely related.

To examine the duplication events of *PLD* genes in cotton, the tandem, and segmental duplications were identified. A total of 28, 28, 4, 3, and 4 segmental gene pairs were identified in *G. hirsutum*, *G. barbadense*, *G. herbaceum*, *G. arboreum*, and *G. raimondii*, respectively; four pairs were ascribed to tandem duplication (containing 2, 2, and 1 tandem gene pairs in *G. hirsutum*, *G. barbadense*, and *G. arboreum,* respectively; Supplementary Table S2). Ka/Ks ratios were calculated to measure the selection pressure on these duplicated gene pairs. Among the five cotton species, 70 of 71 duplicated pairs of *PLD* genes had Ka/Ks ratios <1. This implied that the PLD gene family had undergone intense purifying selection pressure in the course of evolution.

### Tissue-Level Expression of *GhPLD* Genes

To further understand the latent developmental roles of *PLD* genes in *G. hirsutum*, we analyzed the published transcriptomic data for the expression of *PLD* genes in diverse cotton tissues (Supplementary Figure S3). The results showed that all *GhPLD* genes had diverse expression patterns in tissues based on the different developmental stages. Two *GhPLDα* genes (*GhPLD13* and *GhPLD33*) showed higher expression levels in the pistil, bract, ovule, and fiber, while other *GhPLDα* isotypes were expressed at lower levels in these tissues. Most *GhPLDδ* genes including *GhPLD2*, *GhPLD5*, *GhPLD19*, *GhPLD22*, *GhPLD24*, and *GhPLD39* showed selectively higher expression in anther and filament and analogously also had high expression in fiber during initiation (−3 to 0 DPA; Supplementary Figure S3). These results indicated that these *GhPLDδ* isotypes may be involved in anther and fiber development.

### Differential Expression Between *psm5* and W10

Based on the analysis of transcriptomic sequencing data in our laboratory (unpublished data), we found that some members of the PLD gene family had significant differential expression between *psm5* and W10 (Supplementary Figure S4A); nine of these genes were significantly differentially expressed in anthers of *psm5* on the day before flowering (B), while the transcripts of only two *GhPLD* genes (*GhPLD12*, *GhPLD18*) had a significant difference in the binuclear period (A), as compared to the WT (Supplementary Figure S4A and Supplementary Table S4). We speculated that *GhPLD* genes (*GhPLD2*, *GhPLD7*, *GhPLD9*, *GhPLD15*, *GhPLD18*, *GhPLD22*, *GhPLD29*, *GhPLD36*, *GhPLD38*) may play an essential role in the later stages of anther development.

To confirm the differential expression of *GhPLD* genes in the *psm5*, we analyzed and compared the levels of expression of 20 randomly selected *GhPLD* genes in the *psm5* and W10 (WT) using quantitative real-time PCR (qPCR). The results of qPCR indicated that the expression of 10 genes was significantly down-regulated in anthers of *psm5* in the B period as compared to WT; the expression of two genes was significantly up-regulated, while the transcripts of most GhPLDs showed no significant differences in the A period (Supplementary Figure S5). Consistent with transcriptomic data, we validated the potential role of the nine *GhPLD* genes (*GhPLD2*, *GhPLD7*, *GhPLD9*, *GhPLD15*, *GhPLD18*, *GhPLD22*, *GhPLD29*, *GhPLD36*, and *GhPLD38*) in the regulation of anther dehiscence in cotton.

### Co-localization of *GhPLD*s With QTL for Fiber Development

**Figure 2:**
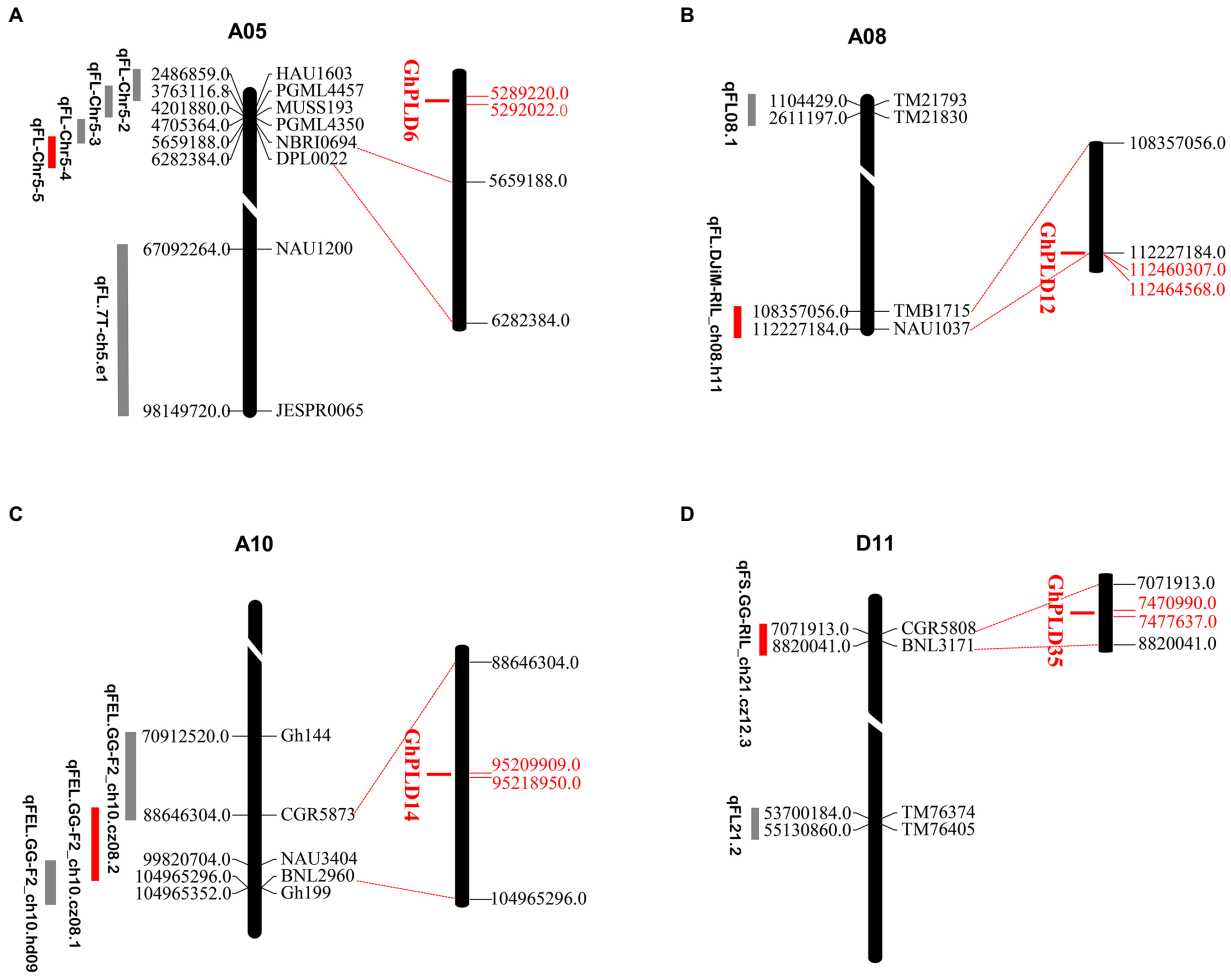
Distribution of co-localized *GhPLD*s on the A05 **(A)**, A08 **(B)**, A10 **(C)**, and D11 **(D)** chromosomes of *G. hirsutum*. The scale indicates the position of *PLD* genes and quantitative trait locus (QTL)-linked markers in the chromosome. The unit is megabase (Mb). QTLs related to fiber length (FL), fiber elongation (FE), and fiber strength (FS) are shown. *GhPLD* genes that co-localize with QTLs of different fiber traits are indicated in red.

Fiber quality traits and yield are quantitative and controlled by numerous QTLs (Jiang et al., 1998). The potential functions of GhPLDs were further examined by mapping the GhPLDs onto the previously reported QTL related to fiber length (FL), fiber elongation (FE), and fiber strength (FS). Four QTL including qFL-Chr5-5 (Ma et al., 2017), qFL.DJiM-RIL_ch08.h11, qFEL.GG-F2_ch10.cz08.1, and qFS.GG-RIL_ch21.cz12 were found to co-localize with *GhPLD6* (PLDα), *GhPLD12* (PLDβ/γ), *GhPLD14* (PLDζ), and *GhPLD36* (PLDβ/γ), respectively. Four *PLD* genes were mapped in the proximity of the QTLs for fiber length, fiber elongation, and fiber strength (Figure 2). Thus, we provided evidence for the potential function of *GhPLD* genes in the fiber development.

### GhPLD2 Protein Is Localized to the Plasma Membrane

**Figure 3:**
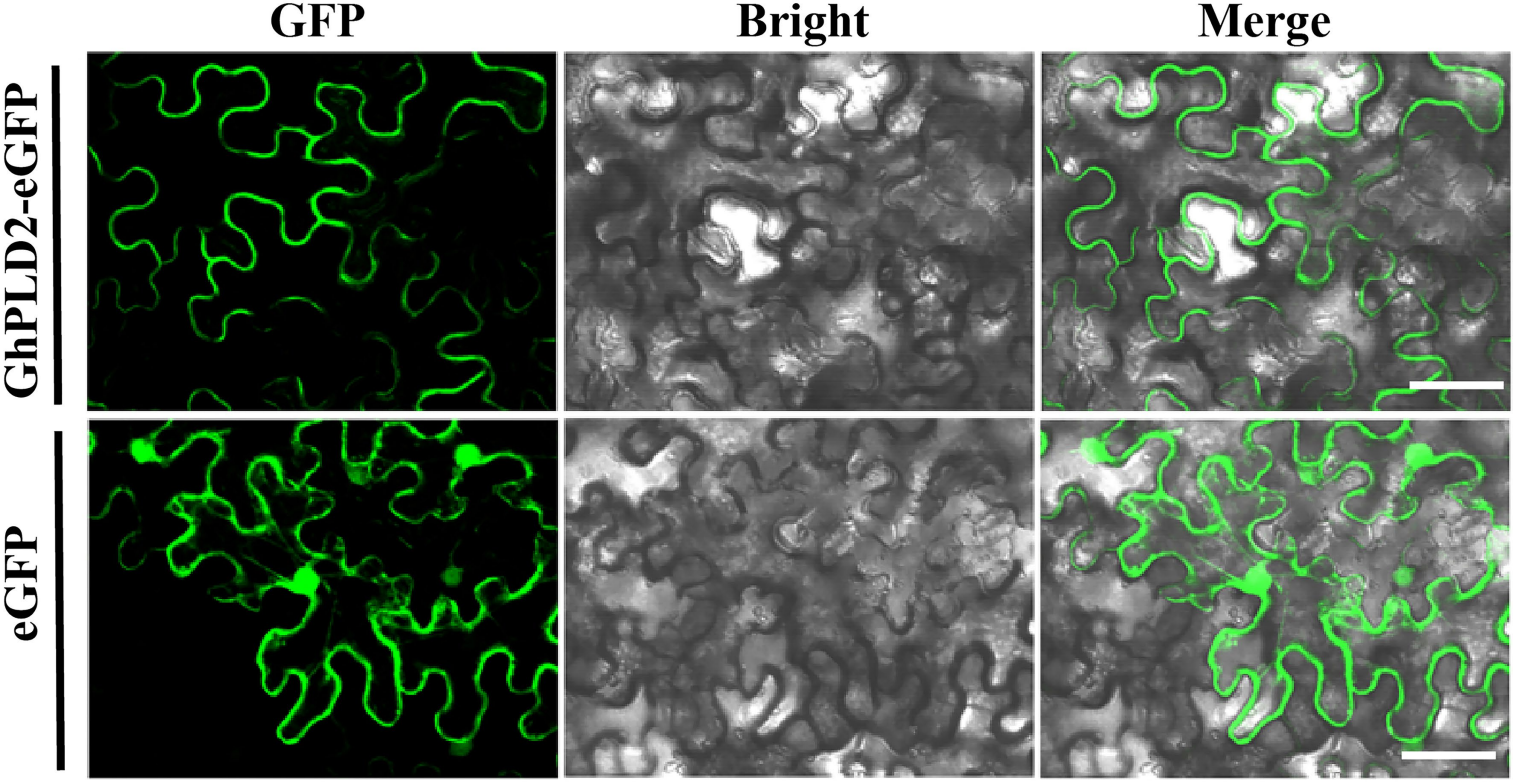
Subcellular localization of GhPLD2 in tobacco (*Nicotiana benthamiana*) leaves. Subcellular localization of GhPLD2. Fluorescence signals from the GhPLD2-eGFP construct were mainly detected at the plasmalemma, while those of empty vector eGFP were detected in the nucleus, cytoplasm, and plasmalemma. Scale bar=50μm.

Based on the significantly higher expression of the *GhPLD2* gene in anther tissue and its most significant differential expression between the *psm5* and W10, we further examined its function during anther dehiscence. The subcellular localization of the GhPLD2 protein was determined using a construct that transiently expressed GhPLD2-enhanced green fluorescent fusion protein (GhPLD2::eGFP) in tobacco leaves. Fluorescence microscopy showed that the empty vector constructs expressed eGFP protein which was localized in the nucleus, cytoplasm, and cell membrane, while the fluorescence signal from the expression of the GhPLD2::eGFP fusion vector was specifically localized to the plasma membrane (Figure 3). We further validated these results using the lipid dye FM4-64 which labels the cell membranes (Supplementary Figure S6). Therefore, GhPLD2 protein was a membrane-localized protein.

### *GhPLD2* May Mediate in Anther Dehiscence

**Figure 4:**
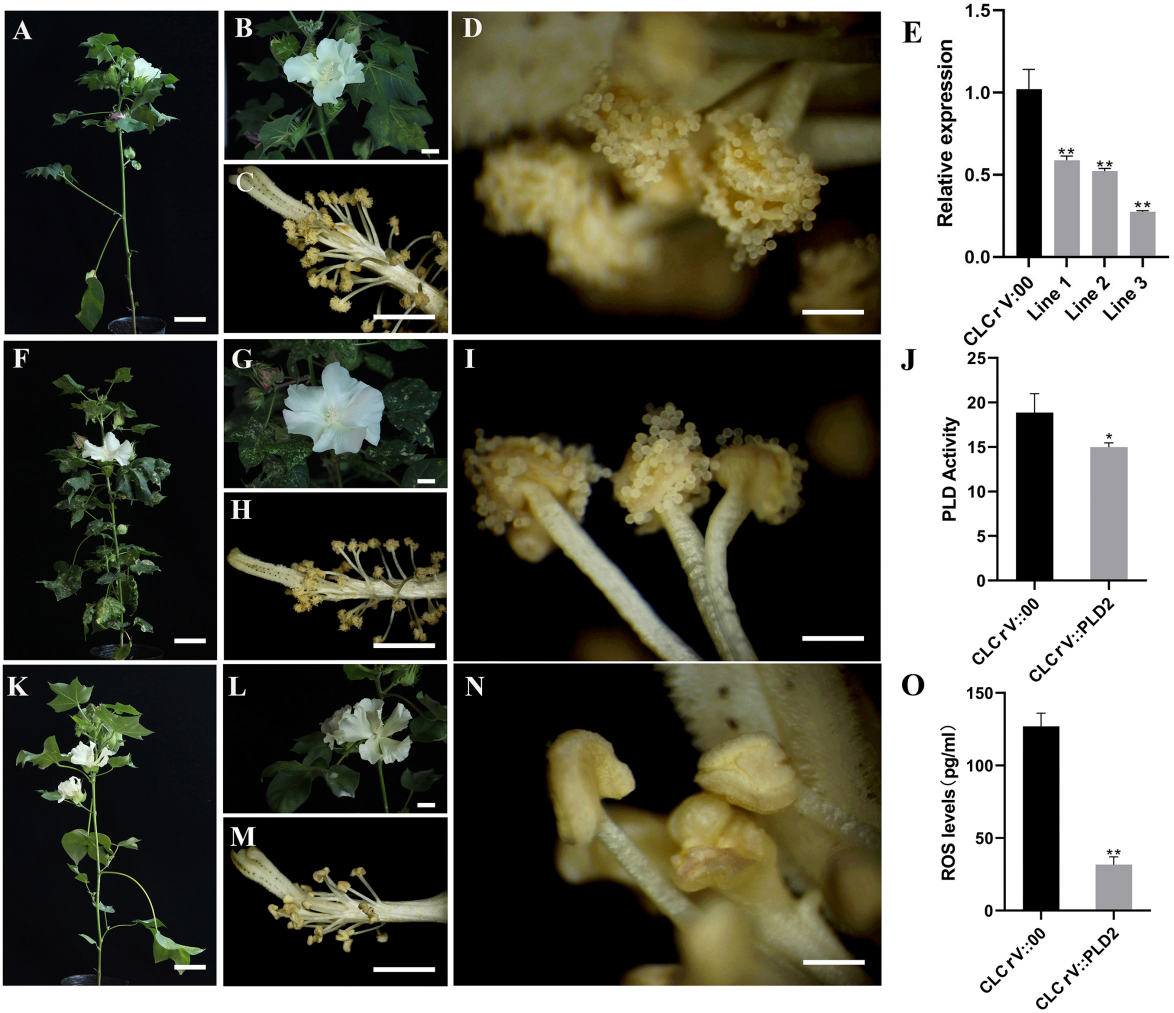
Silencing of *GhPLD2*-induced anther dehiscence defect and reduction in reactive oxygen species (ROS) content in upland cotton. **(A–D)** The cotton plant, flower, and anthers of CLCrV::00 are shown, respectively. **(F–I)** The cotton plant, flower, and anthers of CLCrV::PDS are shown, respectively. **(K–N)** The cotton plant, flower, and anthers of CLCrV::GhPLD2 are shown, respectively. **(E)** Expression levels of *GhPLD2* in CLCrV::00 and CLCrV::GhPLD2 plants. *GhHistone 3* was used as the reference gene for normalization. **(J)** Phospholipase D activity in CLCrV::00 and CLCrV::GhPLD2 plants. **(O)** ROS levels in CLCrV::00 and CLCrV::GhPLD2 plants. Error bars indicate the estimate of standard deviation in three independent replicates. Statistical analyses of experimental data were performed by t-tests. Asterisks showed that the value of *p* indicates significant (^*^ *p*<0.05) or very significant differences (^**^ *p*<0.01) as compared to that of the control. (**A**,**F**,**K**) scale bar=8cm; (**B**,**C**,**G**,**H**,**L**,**M**) scale bar=1cm; (**D**,**I**,N) scale bar=1.5mm.

Male sterility is a hotspot in plants research. In previous studies, the *PLD* genes were shown to have possible involvement in the male sterility by regulating anther dehiscence (Qiu et al., 2007). To verify this possibility for the *PLD* gene in cotton, virus-induced gene silencing (VIGS) assay was used to silence *GhPLD2* expression. Due to *GhPLD2* downregulation, anther indehiscence occurred in CLCrV::GhPLD2 plants (Figure 4 A-N). To further determine the *GhPLD2* gene silencing efficiency, qPCR was used to assess the quantitative expression of *GhPLD2* in both the CLCrV::GhPLD2 and CLCrV::00 cotton plants. Notably, *GhPLD2* expression was significantly reduced in CLCrV::GhPLD2 plants as compared to the CLCrV::00 control plants (Figure 4E). PLD activity in the CLCrV::GhPLD2 plants was also significantly downregulated (Figure 4J). In addition, ROS levels in the anther were also significantly reduced along with a decrease in phospholipase activity (Figure 4O), consistent with late anther development in *psm5* as compared to W10 (Supplementary Figure S4B). These findings suggested that the *GhPLD2* gene may regulate the development of anther dehiscence through its effects on PLD activity and ROS levels.

### *GhPLDs* May Regulate Fiber Initiation

**Figure 5:**
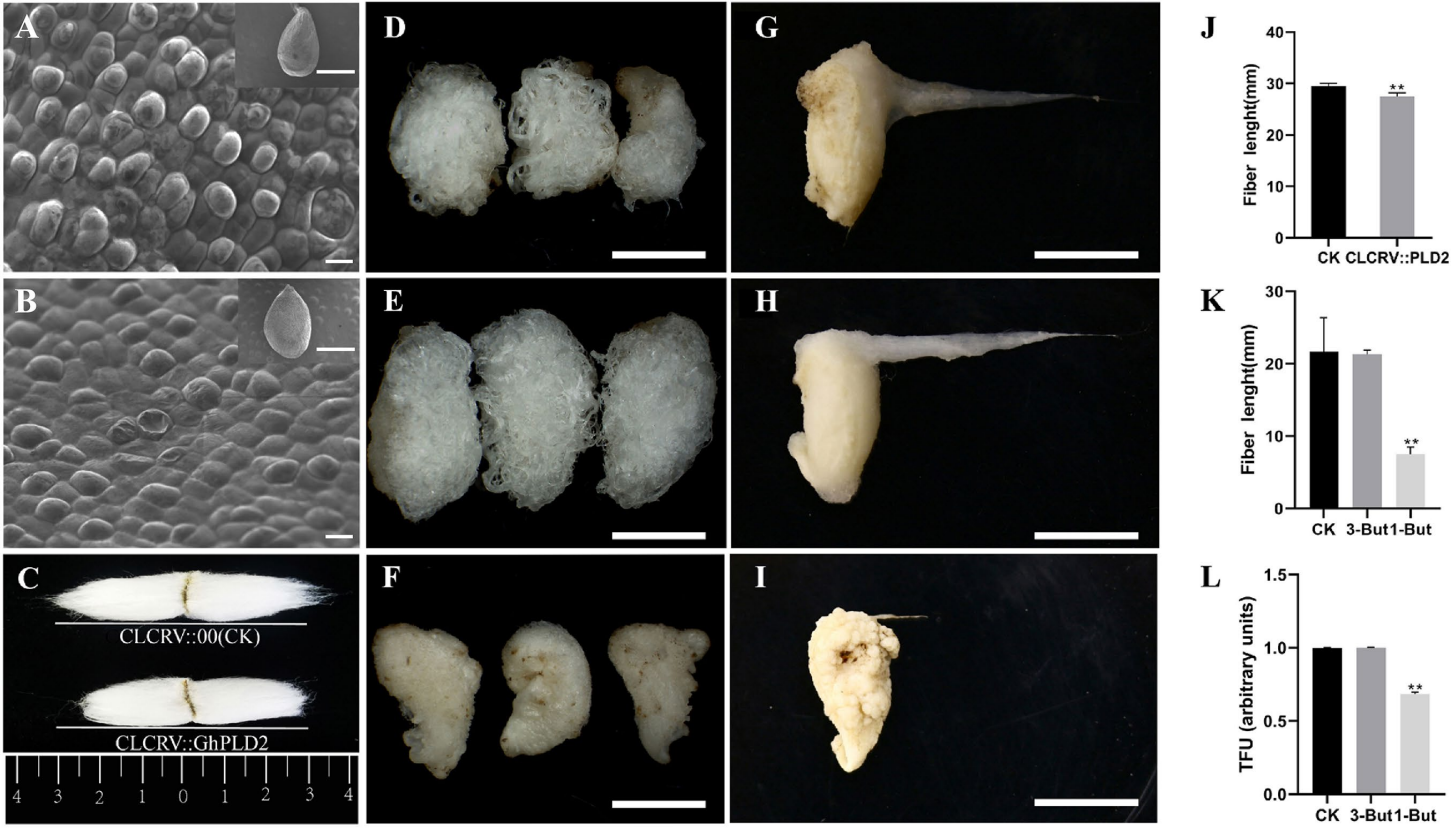
Inhibition of PLD activity affects fiber elongation, and GhPLD2 is involved in fiber initiation and elongation. **(A–C)** Silencing *GhPLD2* inhibited fiber initiation (**A**, ovule of blank control, **B**, ovule of VIGS plant) and significantly reduced fiber length (**C,J**) scale bar=1mm, scale bar=10μm. **(D–I)** Seed and fiber morphological characteristics in the *in vitro* culture after treatment with 1-But **(F,I)**, 3-But **(E,H)**, and sterile water (CK; **D,G**); scale bar=1cm. **(K)** Fiber length in 1-But and 3-But treated cultures. **(L)** Total fiber units (TFUs) in 1-But and 3-But treated cultures. Error bars show the standard deviation estimated using three independent experimental repeats. Statistical analyses of experimental data were performed using *t*-tests. Asterisks show that the values of *p* indicate very significant (^**^ *p*<0.01) differences as compared to the control.

Phospholipase D function has been shown to underlie fiber elongation (Tang and Liu, 2017). To confirm the likely involvement of *GhPLD2* in the regulation of fiber initiation, the protodermal cells of the cotton ovule on the day of anthesis were observed using a scanning electron microscope. We found that ballooning out of protodermal cells was delayed in CLCrV::GhPLD2 plants (Figures 5A,B). Subsequently, mature fiber length was measured in CLCrV::GhPLD2 and CLCrV::00 plants (Figures 5C,J); the mature fiber length of CLCrV::GhPLD2 was significantly lower than that for the CLCrV::00 plant. These results suggested that GhPLDs may play a novel role in fiber initiation in addition to regulation of fiber strength (Tang and Liu, 2017).

Phospholipase D regulates plant growth and development through the generation of PA as a lipid messenger. To evaluate the relationship between PLD, microtubules, and cotton fiber development, the phenotypic observation and measurement of the fiber length and TFUs using *in vitro* cotton ovule cultures were performed at 20 DPA (Figures 5D–I,K,L). The results suggested that the length and TFUs of fiber were disposed by 1-butanol, a specific inhibitor of PA; there was no difference with 3-butanol treatment, as it cannot activate PLD. These results further showed that *GhPLD* genes may regulate microtubule stability by altering fiber initiation and elongation.

## Discussion

### PLD Family in Cotton Expanded During Evolution

We found that the number of *PLD* genes varied among different cotton species, with 40, 40, 21, 21, and 20 genes expressed in *G. hirsutum*, *G. barbadense*, *G. herbaceum*, *G. arboreum*, and *G. raimondii*, respectively. The variability of member of PLD gene in *G. arboreum* was compared with previous reports (Tang et al., 2016b), and this phenomenon may be attributed to the difference reference genomes. We have used the latest genome assemblies for analyses of the PLD family in *G. arboreum* genomes (Huang et al., 2020). Therefore, the new databases undoubtedly facilitated accuracy in the identification of PLD gene family in cotton.

Polyploidy is a feature of genome evolution in allotetraploid cotton that doubled the size of the PLD family due to whole-genome duplication (WGD) and segmental duplication. Segmental duplication events result in a wide dispersion of gene copies (BaumGaten et al., 2003). For example, a total of 12 *PLD* genes one *PLDδ* gene are found in *Arabidopsis*, while the number of *PLDδ* genes in cotton is much higher. There are ten *PLDδ* in allotetraploid cotton species, and five *PLDδ* genes in diploid cotton species. Compared with *A. thaliana*, we found that the expansion of the cotton δ branch in the cotton genome was higher. This phenomenon occurs in other species including *G. max* (Zhao et al., 2012), *B. napus* (Lu et al., 2019), *P. alba*, and *V. vinifera* (Liu et al., 2010). Correspondingly, there are several segmental gene pairs in the δ clade of the PLD family in cotton (Supplementary Table S2). Gene duplication may have played an important role in the evolution of phenotypic novelty within plants. This tendency indicates the specific evolution of the PLDδ branch and expansion of PLDδ isoforms which is necessary for adaptive evolution of plants. This also indicates that segmental duplication might have contributed to the expansion of the cotton PLD gene family. Similarly, the occurrence of tandem replication events has facilitated the expansion of the gene family (Cannon et al., 2004; Leister, 2004). Here, four important tandem duplications were found only in *G. hirsutum*, *G. barbadense*, and *G. arboreum*, which indeed supported the above point of view. In conclusion, our results confirmed that segmental replication and WGD were the main driving forces for the amplification of the PLD gene family in cotton.

### *GhPLD* Genes May Mediate Anther Dehiscence in Cotton

Male sterility is a common phenomenon in flowering plant species, occurring in several crops (Liu et al., 2014; Li et al., 2016; Chen et al., 2020b; Saxena et al., 2020). Flowering plants require normal anther dehiscence to ensure timely pollen release at fruit-setting stages. The tissue-specific expression analysis of *PLD* genes showed that most *PLD*s, especially *GhPLDδ*s, had higher expression in the anther and filament (Supplementary Figure S4), consistent with previous studies (Tang et al., 2016a). Furthermore, a recent study also suggests that the PLD signaling pathway is mostly enriched in the late developmental stage of the anther in cotton (Chen et al., 2018). A new photosensitive cotton mutant, *psm5*, exhibits male sterility resulting from the absence of anther dehiscence in long-day conditions. qPCR analyses were performed, and the results independently confirmed the differential expression of *PLD* genes in the late stage of anther development in *psm5* as compared to the WT. Among these differentially expressed genes, *GhPLD*s belonging to the δ clade accounted for the majority proportion. *GhPLD13* (α) and *GhPLD39* (δ) were upregulated in *psm5*, while the 10 other *GhPLD*s were downregulated (Figure 5). The results indicated that *GhPLDδ*s may be involved in anther dehiscence, and may underlie male infertility in *psm5*. In this study, *GhPLD2* was chosen for subsequent functional analyses due to its high expression in anther tissue and significant differential expression in *psm5*. Silencing the gene expression of *GhPLD2* by VIGS further validated that *GhPLD2* was indeed involved in anther dehiscence and was a positive regulator. Although the efficiency of silencing was only about 40–70%, and the flowers with non-dehiscent anthers accounted for approximately 22%. This implied that even a partial loss of *GhPLD2* gene function could have a severe impact on plant development.

Furthermore, the ROS levels in anther tissues significantly decreased along with the reduction in PLD activity in CLCrV::GhPLD2 cotton plants. ROS production is catalyzed by NADPH oxidase (Livanos et al., 2012a); AtPLDα1 and phosphatidic acid (PA) increase the activity of NADPH oxidase and ROS production (Zhang et al., 2009). Based on a comprehensive analysis of these results, we speculated that GhPLD2 regulates NADPH oxidase activity to promote ROS production, and the silencing of *GhPLD2* in CLCrV::GhPLD2 plants may alter ROS accumulation to induced anther indehiscence.

### *GhPLD* Genes May Control Fiber Growth in Cotton

In this study, the identification of QTLs associated with *GhPLDα*s, *GhPLDβ*/*γ*s, and *GhPLDζ*s provided strong evidence for the role of *PLD* genes in the fiber development. A previous study reported PLDα protein expression in an elongating cotton fiber sample (Yang et al., 2008). The fiber elongation role of *GhPLDa1* in the α branch has been reported for upland cotton (Tang and Liu, 2017). Here, tissue-specific expression analysis suggests that *GhPLD2*, *GhPLD5*, *GhPLD19*, *GhPLD24*, *and GhPLD38* genes, belonging to the δ branch, could not only regulate fiber elongation but may also be involved in fiber initiation.

Phospholipase D regulates plant growth and development through the generation of a lipid messenger, PA. 1-butanol, a specific inhibitor of PLD-dependent production of the signaling molecule PA, disrupts the organization of interphase cortical microtubules (Gardiner et al., 2003); 3-butanol can be used as a control for any non-specific butanol effect as it has no role in PLD activation (Munnik et al., 1995). The results of *in vitro* cotton ovules cultures showed that the length and TFUs of fiber dealt with 1-butanol were significantly inhibited, and this may be attributed to the effects of 1-butanol on the conformation of microtubules in fibroblasts. PLD serves as a dynamic membrane linker for microtubules, and its activation induces microtubule depolymerization and rearrangement, which help cells growth adjustment, division, and stimulus-response requirements owing to its different conformations (Whittaker and Triplett, 1999; Dhonukshe and Gadella, 2003; Li et al., 2004; Zhang et al., 2017). Taken together, the results of the VIGS experiment, it was suggested that *GhPLD* genes may regulate microtubule stability, and thus impact both fiber initial and elongation (Supplementary Figure S7).

## Conclusion

In conclusion, a total of 40, 40, 21, 21, and 20 *PLD* genes were identified in *G. hirsutum*, *G. barbadense*, *G. herbaceum*, *G. arboreum*, and *G. raimondii*, respectively. Their phylogenetic classifications, gene structures, motifs, and gene duplications were analyzed. The *PLD* genes in the same evolutionary branches were found to share similar motifs and gene structures. Our analysis indicated that WGD and segmental duplication events were the main driving forces underlying *PLD* gene amplification and that purifying selection played a major role in the evolution of the *PLD* gene family.

Based on the transcriptomic analysis, higher expression of *GhPLDδ* genes including *GhPLD2*, *GhPLD5*, *GhPLD19*, *GhPLD24*, and *GhPLD39* was found during the fiber initiation (−3 to 3 DPA) and elongation (5–20 DPA) periods. VIGS experiments showed that *GhPLD2* had a positive impact on fiber initiation and elongation. qPCR results indicated that differential expression of some *GhPLD* genes may underlie male sterility in cotton. Silencing of the *GhPLD2* gene assays indicated that GhPLD2 may be involved in ROS production by NADPH oxidase, and thus may positively regulate anther dehiscence. In conclusion, our study provides functional insights into the roles of the PLD gene family in male sterility and fiber development in cotton.

## Data Availability Statement

The original contributions presented in the study are included in the article/Supplementary Material, further inquiries can be directed to the corresponding authors.

## Author Contributions

CM: conceptualization, methodology, software, and writing. QZ: methodology and data curation. JL and KQ: software and visualization. SF: visualization and investigation. QM and CZ: reviewing and editing. All authors contributed to the article and approved the submitted version.

## Funding

This work was supported by the Major Research Plan of the National Natural Science Foundation of China (Grant no. 31690093), the National Natural Science Foundation of China (Grant no. 31701474), and Agricultural Science and Technology Innovation ProGrm of Chinese Academy of Agricultural Sciences.

## Conflict of Interest

The authors declare that the research was conducted in the absence of any commercial or financial relationships that could be construed as a potential conflict of interest.

## Publisher’s Note

All claims expressed in this article are solely those of the authors and do not necessarily represent those of their affiliated organizations, or those of the publisher, the editors and the reviewers. Any product that may be evaluated in this article, or claim that may be made by its manufacturer, is not guaranteed or endorsed by the publisher.

## Supplementary Material

The Supplementary Material for this article can be found online at: https://www.frontiersin.org/articles/10.3389/fpls.2021.728025/full#supplementary-material

Supplementary Figure S1 Phylogenetic relationships and gene structure of the PLDs in *Gossypium hirsutum*, *G. barbadense*, *G. herbaceum*, *G. arboreum*, *G. raimondii*, and *A. thaliana*. **(A)** The phylogenetic tree was constructed based on the full-length protein sequences of PLD using MEGA 7 software. **(B)** Exon–intron structures of PLDs. Green boxes indicate exons; yellow boxes indicate untranslated regions; black lines indicate introns.

Supplementary Figure S2 Collinear analyses for *G. hirsutum*, *G. barbadense*, *G. herbaceum*, *G. arboreum*, and *G. raimondii*. **(A)** Collinear analysis for the A-genome of allotetraploid cotton species (*G. hirsutum* and *G. barbadense*) and diploid cotton species (*G. herbaceum* and *G. arboreum*). **(B)** Collinear analysis for D-genome of allotetraploid cotton species and *G. raimondii*. Orange lines depict the collinearity of the *PLD*s between the genomes of two allotetraploid cotton species; green lines depict the collinearity of the *PLD*s for the genomes of allotetraploid cotton and diploid cotton; gray lines in the background depict the collinear blocks within the different genomes, while other colored lines highlight the syntenic *PLD* gene pairs.

Supplementary Figure S3 The expression patterns of the *GhPLD*s according to a common database in different tissues and organs and during fiber development. **(A)** The expression patterns of the *GhPLD*s in different tissues including anther, filament, pistil, bract, sepal, petal, torus, root, leaf, and stem. **(B)** The expression profiles of the *GhPLD*s at −3, 0, 1, 3, 5, 10, 15, 20, and 25 day post-anthesis (DPA) ovules and 10, 15, 20, and 25 DPA fibers.

Supplementary Figure S4 **(A)** The expression profiles of *GhPLD* genes in W10 (WT) and *psm5* based on RNA-seq data. Transcriptome data are summarized in Supplementary Table S4. Data are presented as log_2_fold change. **(B)** Mitochondrial reactive oxygen species (ROS) production rate in anthers of *psm5* and W10. The first stage A is a binuclear phase of the microspore, the early flowering stage (flower bud length, ~10mm). The second stage B is 1day before flowering. Each experiment was performed with three biological repeats. The values are presented as the mean±SDs of the three replicates. Value of *p* was calculated based on the Student’s *t*-tests.

Supplementary Figure S5 Differential expression profiles of the *GhPLD*s in W10 (WT) and *psm5*. The first stage A is a binuclear period of the microspore, the early flowering stage (flower bud length, ~10mm). The second stage B is 1day before flowering. The values are presented as the mean±SDs of three replicates. Values of *p* were calculated using the Student’s *t*-tests. ^**^ denotes significance at *p*<0.01 as compared to the WT. The details of the primer sequences are listed in Supplementary Table S3.

Supplementary Figure S6 Re-validation of subcellular localization of GhPLD2 in tobacco (*Nicotiana benthamiana*) leaves. The results in Figure 3 were re-confirmed using the lipid dye FM4-64 (Marker) which labels the cell membranes. Scale bar=50μm.

Supplementary Figure S7 Model of GhPLD2 regulating anther dehiscence and fiber development in cotton. To visually present the function of the *GhPLD2* gene, a model depicting the mechanisms underlying *GhPLD2* gene regulation of anther dehiscence, fiber initiation, and fiber elongation was constructed. GhPLD2 positively promotes ROS production to maintain normal anther dehiscence and fiber development.

## Footnotes

^1^ http://www.cottonfgd.org/

^2^ https://www.cottongen.org/

^3^ http://www.arabidopsis.org/

^4^ http://pfam.xfam.org/

^5^ http://smart.emblheidelberg.de/

^6^ https://www.ncbi.nlm.nih.gov/Structure/cdd/wrpsb.cgi

^7^ https://www.expasy.org/

^8^ http://www.cbs.dtu.dk/services/CPHmodels/

^9^ https://cottonfgd.org/sequenceserver/

^10^ https://www.ncbi.nlm.nih.gov/sra/?term=PRJNA248163

## Abbreviations

PLD: Phospholipase D
PA: Phosphatidic acid
At: *Arabidopsis thaliana*
Gh: *Gossypium hirsutum*
Gb: *Gossypium barbadense*
Ga: *Gossypium arboretum*
Ghe: *Gossypium herbaceum*
Gr: *Gossypium raimondii*
*psm4*: *Photoperiod sensitive male sterility mutant 4 of upland cotton*
*psm5*: *Photoperiod sensitive male sterility mutant 5 of cotton*
VIGS: Virus-induced gene silencing
ROS: Reactive oxygen species
