# Supplementary figures and images for: Genome-Wide Analysis of the Phospholipase D Family in Five Cotton Species, and Potential Role of GhPLD2 in Fiber Development and Anther Dehiscence
Source: Front Plant Sci. 2021 Oct 1;12:728025. doi: 10.3389/fpls.2021.728025 (PMC8517146; doi:10.3389/fpls.2021.728025)

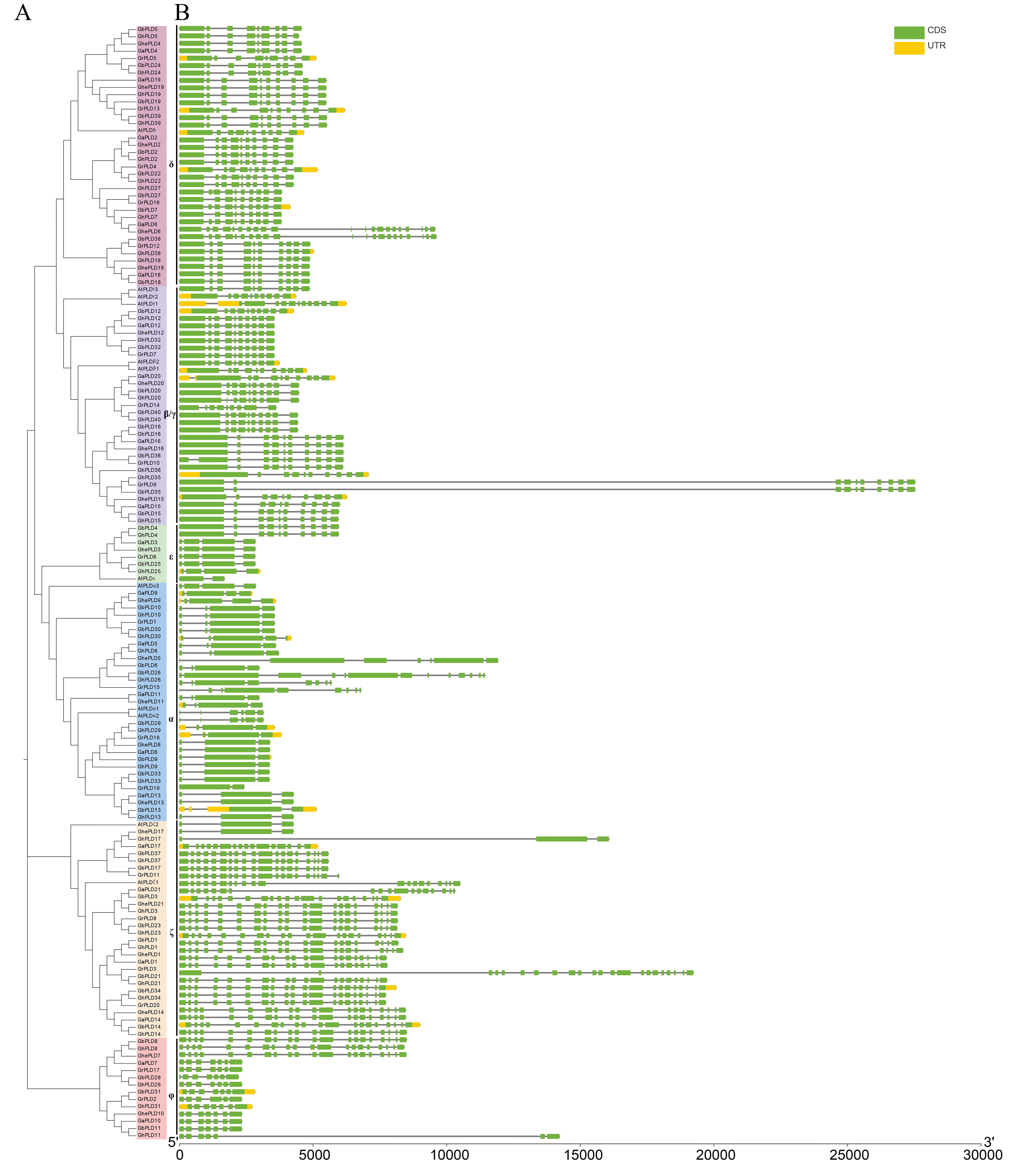

Supplement: Supplementary file 1 [file Data_Sheet_1.zip › Data Sheet 1/Addational file/Figure S1.jpg]

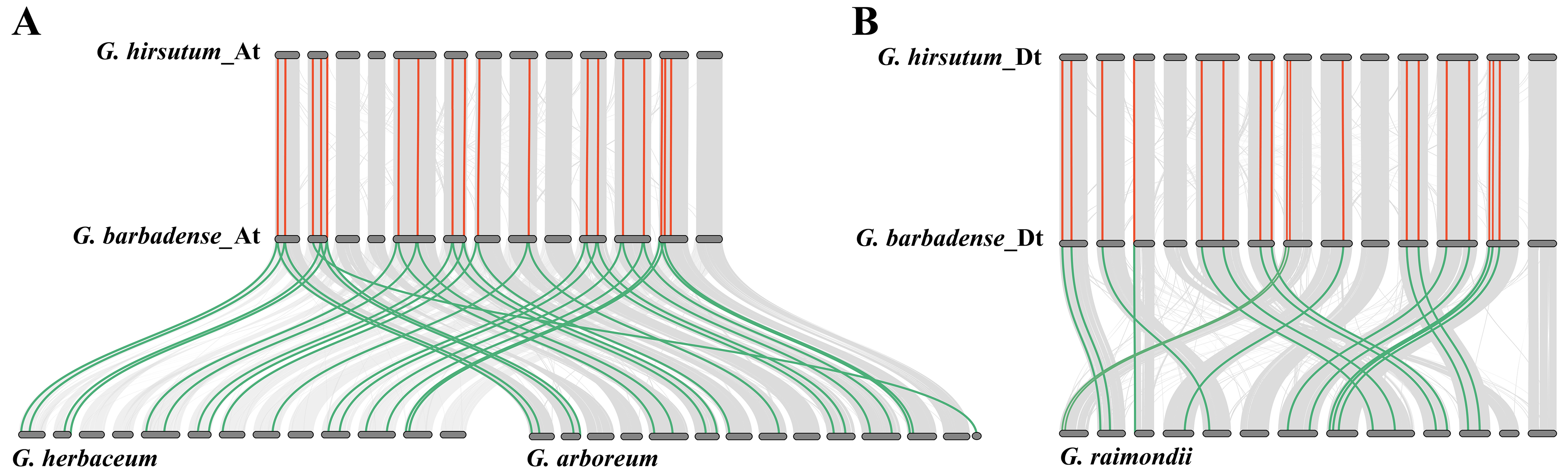

Supplement: Supplementary file 1 [file Data_Sheet_1.zip › Data Sheet 1/Addational file/Figure S2.jpg]

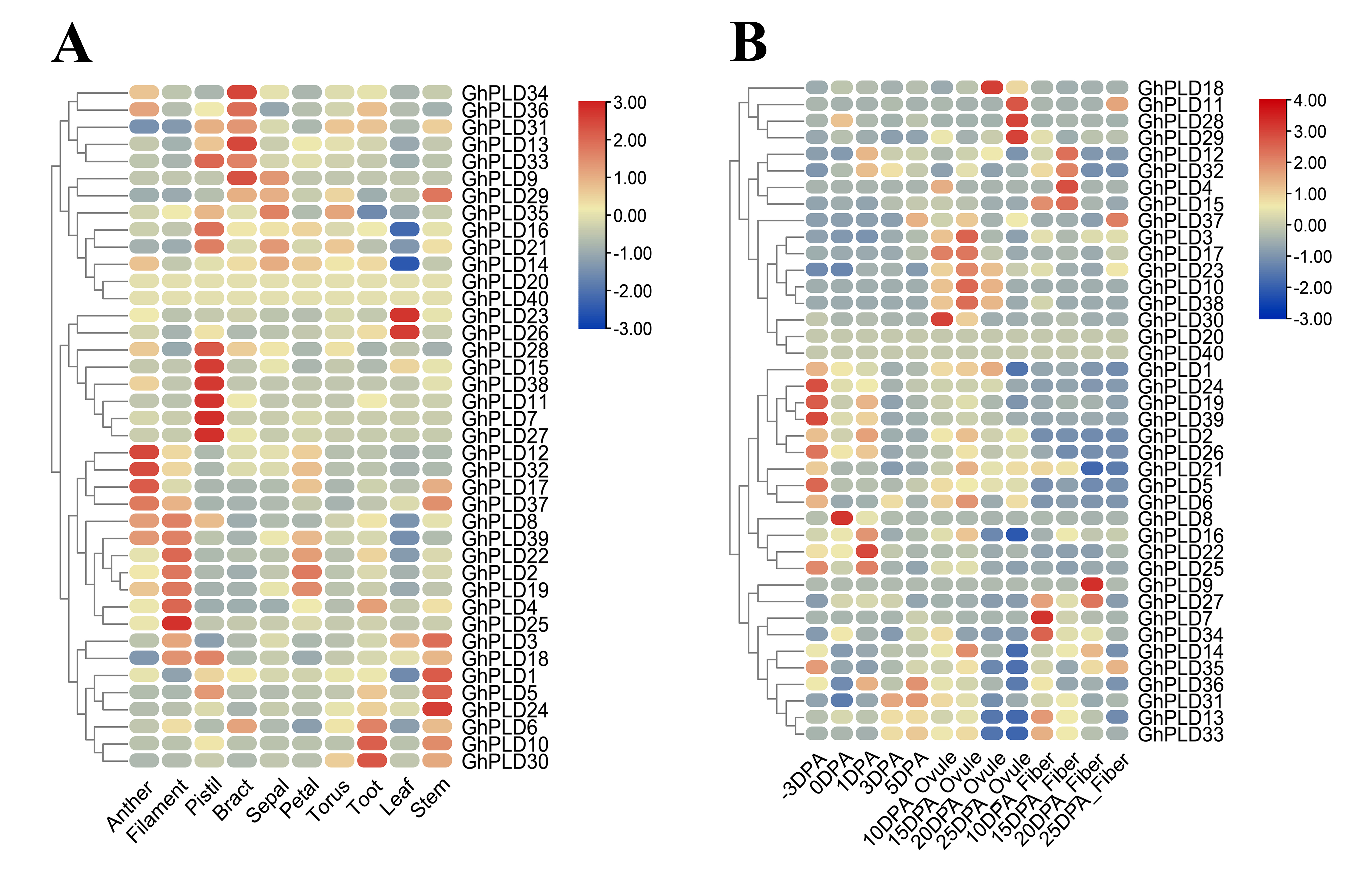

Supplement: Supplementary file 1 [file Data_Sheet_1.zip › Data Sheet 1/Addational file/Figure S3.jpg]

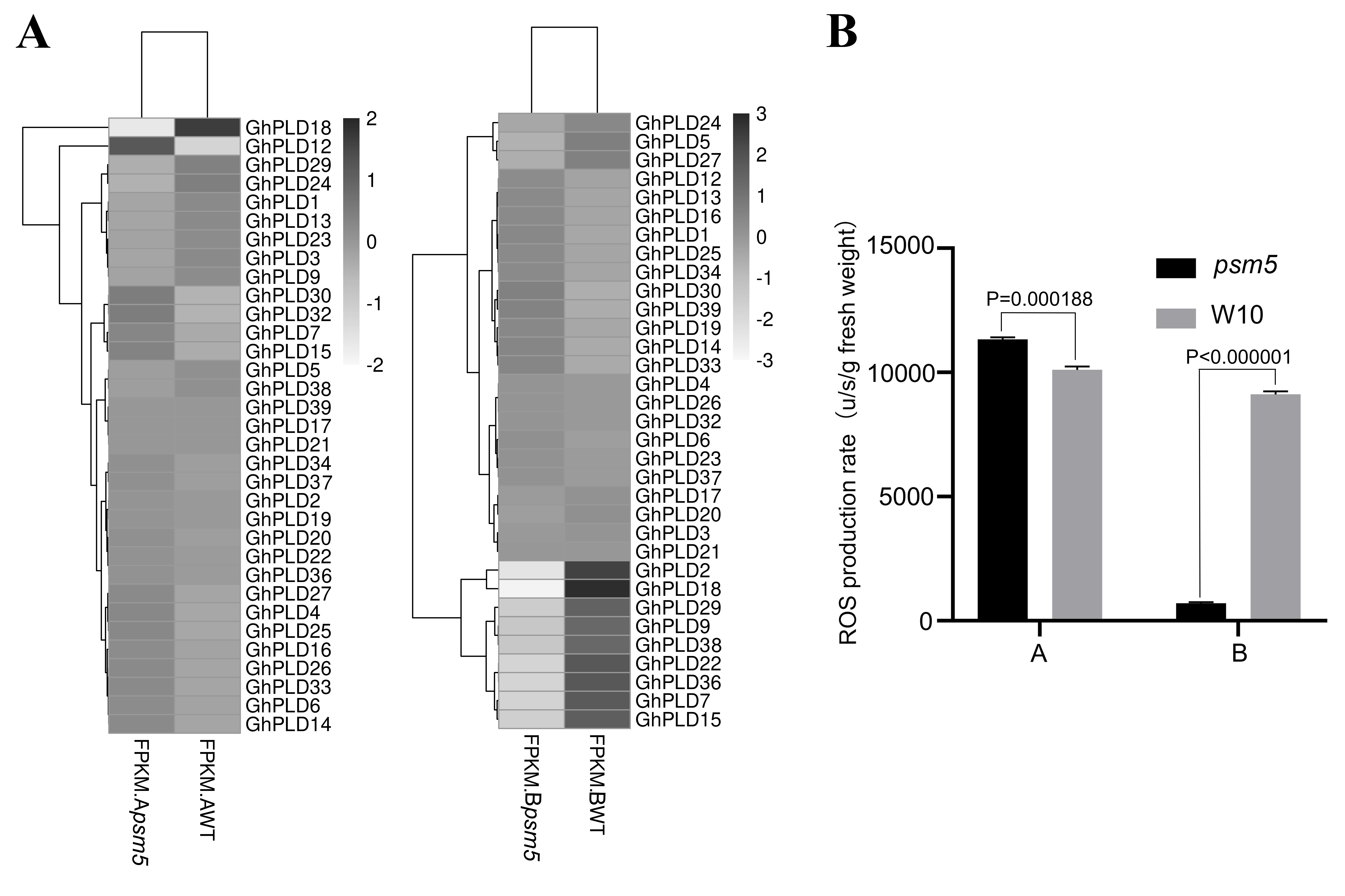

Supplement: Supplementary file 1 [file Data_Sheet_1.zip › Data Sheet 1/Addational file/Figure S4.jpg]

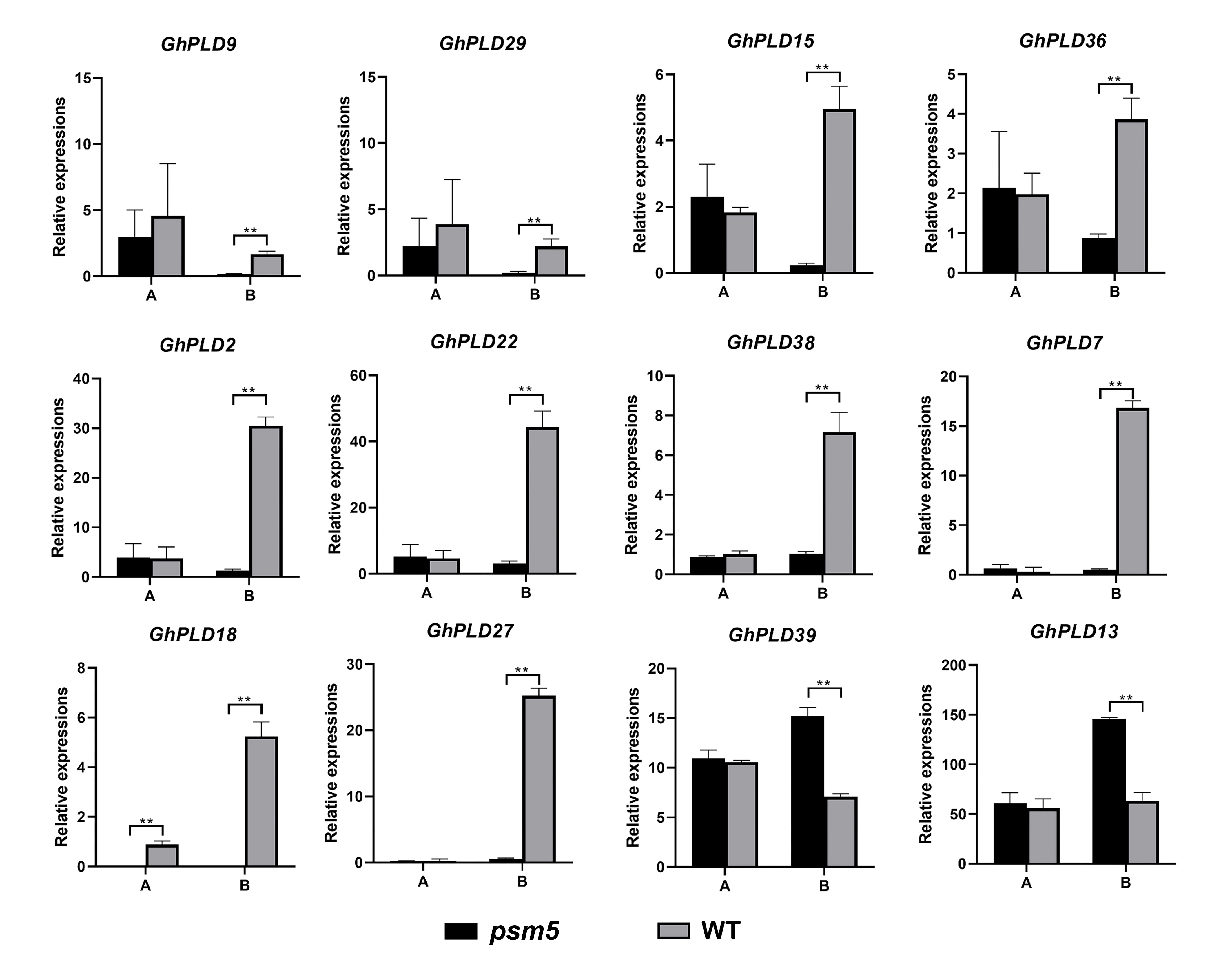

Supplement: Supplementary file 1 [file Data_Sheet_1.zip › Data Sheet 1/Addational file/Figure S5.jpg]

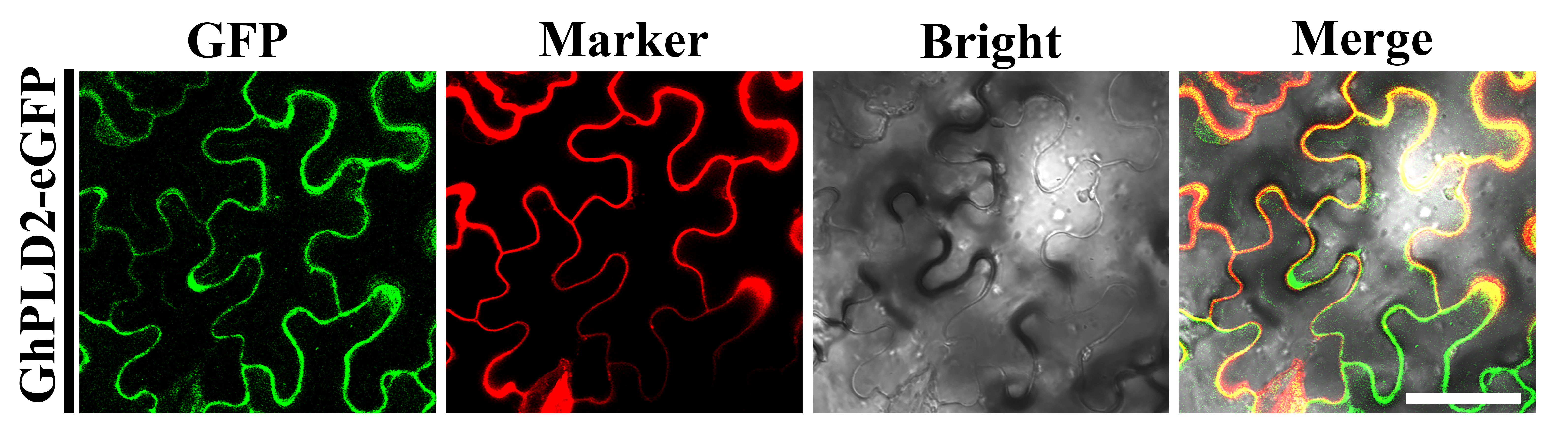

Supplement: Supplementary file 1 [file Data_Sheet_1.zip › Data Sheet 1/Addational file/Figure S6.jpg]

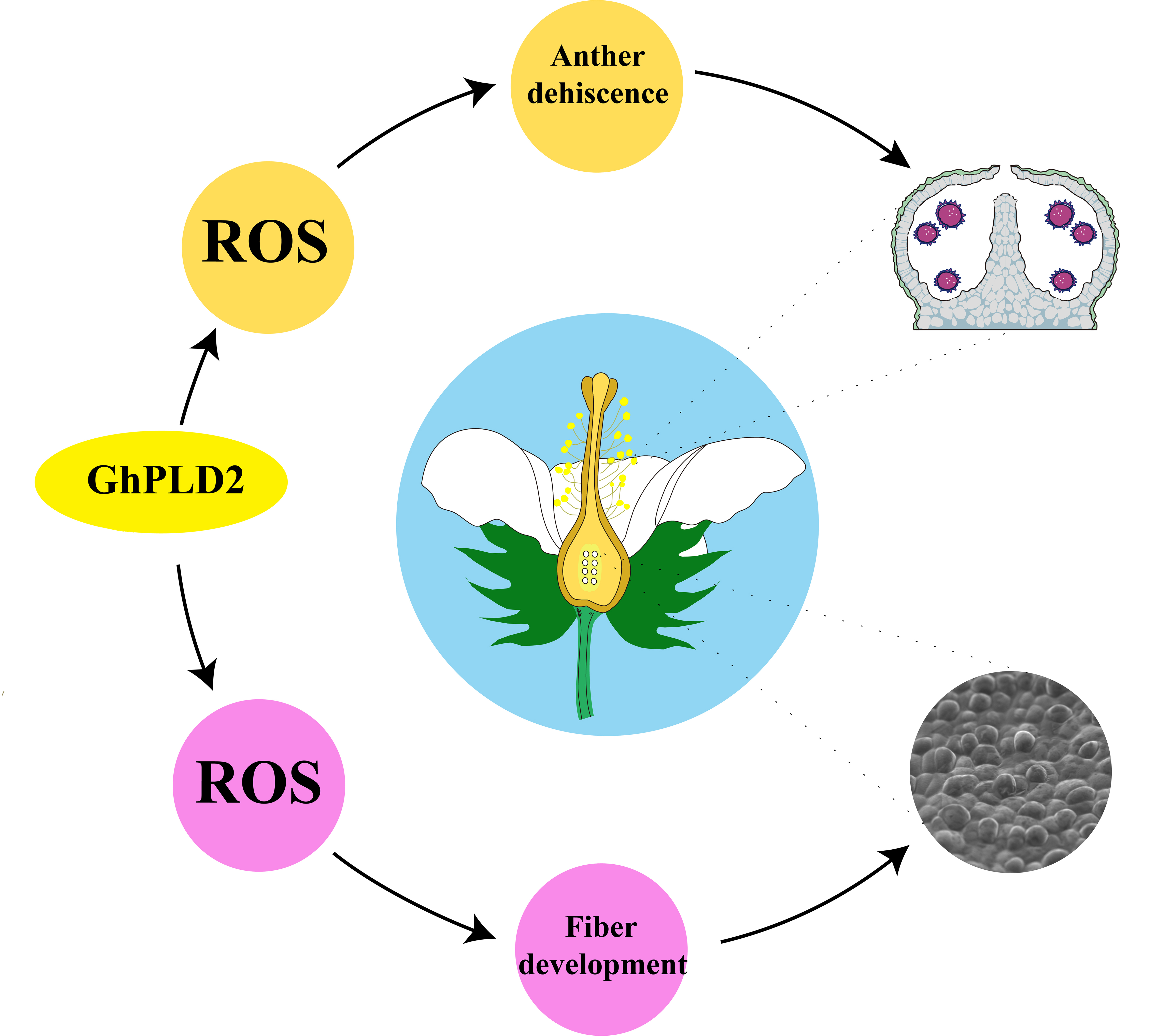

Supplement: Supplementary file 1 [file Data_Sheet_1.zip › Data Sheet 1/Addational file/Figure S7.jpg]
